# Mission of Mercy emergency dental clinics: an opportunity to promote general and oral health

**DOI:** 10.1186/s12889-018-5792-z

**Published:** 2018-07-13

**Authors:** Devlon N. Jackson, Susan Passmore, Craig S. Fryer, Jie Chen, Dushanka V. Kleinman, Alice M. Horowitz, James Butler, Mary A. Garza, Sandra C. Quinn, Stephen B. Thomas

**Affiliations:** 1 Maryland Center for Health Equity, School of Public Health, University of Maryland, 4200 Valley Drive, College Park, MD USA; 2 Department of Health Services Administration, School of Public Health, University of Maryland, 4200 Valley Drive, College Park, MD USA; 3 Department of Behavioral and Community Health, School of Public Health, University of Maryland, 4200 Valley Drive, College Park, MD USA; 4 Department of Epidemiology and Biostatistics, School of Public Health, University of Maryland, 4200 Valley Drive, College Park, MD USA; 5 Center for Health Literacy, School of Public Health, University of Maryland, 4200 Valley Drive, College Park, MD USA; 6 Department of Family Science, School of Public Health, University of Maryland, 4200 Valley Drive, College Park, MD USA

**Keywords:** Public health, Community health, emergency dental clinics, Health equity, Oral health, Mission of mercy

## Abstract

**Background:**

Mission of Mercy (MOM) emergency dental clinics are a resource for populations lacking access to dental care. We designed a MOM event incorporating health equity components with established community partners who shared a common vision of addressing the oral health, physical health, and social service needs of Maryland and Washington, DC area residents. Although studies have explored associations between oral and chronic health conditions, few studies to our knowledge have examined the relationship between these conditions and receipt of dental services. Therefore, this study explored these associations and the opportunity for better care coordination.

**Methods:**

Oral health data from the 2014 Mid-Maryland Mission of Mercy and Health Equity Festival event was analyzed. A descriptive analysis assessed frequencies and percentages of participant sociodemographics characteristics, oral health and chronic disease risk(s), and dental services delivered. Chi-square tests and multivariate logistic regression were conducted to determine the associations between 1) oral health and chronic disease risk(s) and dental services; and 2) oral health and chronic disease risk(s) and participant characteristics.

**Results:**

Approximately 66.2% (*n* = 666) of the 1007 participants had one or more chronic conditions and/or risk factors (diabetes, high blood pressure, and tobacco use). These individuals had a significantly higher likelihood of receiving an oral surgery procedure (specifically, tooth extraction) (only one condition/risk: OR = 2.40, 95%, CI = 1.48–3.90, *p* < .001; two conditions/risks: OR = 3.12, 95% CI = 1.78–5.46, *p* < .001).

**Conclusion:**

The 2014 Mid-Maryland Mission of Mercy emergency dental clinic attracted people with risk factors for oral and chronic diseases. Those with one or more risk factors were more likely to receive oral surgery (specifically, tooth extraction). These findings strongly suggest that organizers of MOM emergency dental clinics include wrap-around primary care, health promotion and disease prevention services along with provision of dental services. While such events will not solve the general and oral health challenges of participants, we believe they provide an opportunity to provide basic preventive services. These findings also present an opportunity to inform planning for future MOMs and emphasize the importance of using these public health events to create linkages with other services to support follow-up and care coordination.

## Background

There is a growing body of evidence that having poor oral health is linked to chronic diseases such as diabetes [1–3] and heart disease [4, 5]. In addition, tobacco use is a risk factor for periodontal diseases and oral cancers [6, 7]. The primary aim of this study was to explore the relationship between chronic diseases and the types of dental services performed at a Mission of Mercy (MOM) emergency dental clinic.

Mission of Mercy (MOM) emergency dental programs have become a frequent choice of last resort for far too many people in need of dental care. These community-based, voluntary dental care settings occur annually throughout the country and attract large numbers of individuals seeking care for dental-related pain and disease. Many individuals seek care from periodic dental events such as MOMs due to the economic and policy issues related to the provision of oral health services for underserved populations. These issues include lack of funds to pay for adult dental services [8, 9], being uninsured and underinsured [10], and Medicaid dental benefits that vary from state to state [11] and seldom cover actual cost of dental care. Some MOMs also provide medical care and health education services in addition to traditional dental care services [12] in an attempt to address other health needs of the MOM attendees.

In 2014, the University of Maryland Center for Health Equity (M-CHE) in collaboration with Catholic Charities of the Archdiocese of Washington, DC, and the Maryland State Dental Association organized a two-and-a-half-day MOM event. The event included a new component for MOM programs in the state, a Health Equity Festival (HEF) comprised of primary care medical screenings, health education and navigation support for better care coordination. This was M-CHE’s first experience conducting a MOM event and the partnership was an opportunity to extend activities of critical relevance to achieving health equity and improving population health by providing the community with comprehensive services that addressed their immediate dental needs, as well as other related healthcare needs. Thomas et al. [13], describe how MOM participants seeking emergency dental services arrive with risk factors for other chronic disease typically cared for in primary medical care settings. However, given that MOM events are infrequent, time-limited, and focused predominantly on dental care, the capacity to provide additional primary care and social services is challenging. As part of the collaborative planning with MOM partners, including local hospitals, we created an environment that supported coordinated dental triage and treatment and added value with a complementary Health Equity Festival (HEF) dedicated to medical screening and public health education. The HEF included partnerships with non-profit, private sector, hospital and academic organizations and aimed to provide comprehensive wrap-around services. Health services were provided, including: HIV testing; carbon monoxide testing, body composition measurement, vision screening, flu vaccinations, nutrition education, oral health education, and legal consultations.

Recently, the *Communities in Action: Pathways to Health Equity* report [14] provided a conceptual model informed by prior models as a guide for practitioners and community members developing effective programs aimed at achieving health equity. The model includes three key elements that are necessary when implementing a “community-driven” initiative in order to be effective in addressing health inequities: 1) create a shared vision and value of health equity, 2) increase community capacity to shape health outcomes, and 3) foster multi-sector collaboration [14]. Although this report was published after our 2014 MOM event, we actually applied these same concepts in the design and operation of the MOM program based on our experience and lessons learned from prior community-engaged programs that we have implemented. For example, we have more than 15 years of experience developing and sustaining partnerships with other entities in an effort to create effective and comprehensive community wellness [15–17]. Furthermore, the HEF built on the established community partnerships that shared a common vision of addressing needs of underserved populations. Together with these MOM partners, we organized the HEF to address those health needs documented by county and state needs assessments, and in the literature [10, 18]. We provided these additional services so MOM participants could be connected to essential networks within the community, which they may not have had access to prior to attending the MOM.

Although previous studies have explored the relationship between oral health conditions and chronic conditions [1–5, 7], few studies to our knowledge have examined the relationship between the receipt of dental services at a MOM event and presence of risk behaviors and chronic conditions. This study expands on our prior publication of the 2014 Mid-Maryland MOM and HEF, by examining multiple chronic risks/conditions rather than only individual risks/conditions (e.g. diabetes) and the relationship with the type of dental services provided.

## Methods

### Setting

Nationally, MOM events were established to address imminent dental care needs. A comprehensive planning team addressed volunteer and participant recruitment, equipment and supplies, triage and health record documentation, liability insurance, treatment management and referrals among other tasks. Recruitment of licensed provider volunteers was led by the Maryland State Dental Association, and non-clinical support volunteers were recruited and managed by Catholic Charities. The event attracted more than 2000 individuals. Staffing this event involved approximately 1462 volunteers, 560 of whom were oral health professionals (dentists, hygienists, assistants) [13].

When planning our two and half day MOM, we wanted to maintain the integrity of the national MOM events and enhance ours with the implementation of the HEF. The first day, Thursday, was a half-day pre-screening event for individuals who received referrals from local community programs due to their emergency dental care needs. This pre-screening made it possible to address those with the most unmet dental care needs at the outset of the opening of the event. The MOM dental program opened its doors at 7:00 am on Friday and Saturday for participant registration and medical triage. Participant registration included check-in, medical assessment of health history and current medications, and medical triage: blood pressure screenings, and blood glucose testing, and, if necessary, repeated blood pressure screenings. Participants then were escorted to dental triage to determine primary dental care issues and planned treatment.

These MOM attendees were encouraged to visit the HEF vendors before or after receiving their dental treatment. Unfortunately, due to demand, limited time, and volunteer staff, we were able to only serve 1018 participants during the two-and-a-half-day event.

### Data collection

Data were collected by one of our Mid-Maryland MOM partners, *ZystemsGO*, in their secured HIPAA regulated technology-based dental record system, DentaleShare. The University of Maryland Institutional Review Board (IRB) determined that our examination of the de-identified dataset did not require IRB approval for a secondary data analysis. Study protocols and procedures were approved by the University of Maryland Institutional Review Board. We used the following measures from the dental records: sociodemographics; medical history; medications; dental care issue; and treatment received. Of the 1018 oral health medical records, 1007 of them were complete and extracted post the event from the system for the purpose of this study. Sociodemographic and health history data were self-reported utilizing a patient intake form (questionnaire) completed during the registration stage and the medical pre-screening triage stage.

### Data measures

#### Sociodemographics

Demographic data included race, ethnicity, sex, and age collected during the registration stage. Race was categorized as white, black, Hispanic, other, and unreported – i.e., individuals who did not report their race and/or ethnicity. Age was categorized into four groups: 18–34, 35–49, 50–64, and 65 years and older. Sex was categorized as male or female.

#### Dental services

To document the range and distribution of dental services provided, we categorized type of services received as: 1) preventive dental services and 2) dental treatment services. Preventive dental services were subcategorized into oral hygiene instruction, fluoride varnish, and adult prophylaxis. Dental treatment services were subcategorized into full mouth debridement, restorative, endodontics, or oral surgery (tooth extractions). For purposes of analyzing the association between chronic conditions and dental services received, we categorized the variable “preventive only” to include all the preventive services (i.e., oral hygiene instruction, fluoride varnish, and adult prophylaxis) rather than individually analyzing each service because the sample size was not robust enough to support chi-square analysis. In addition, this preventive variable is mutually exclusive to the dental treatment service variable because we wanted to determine if receiving no treatment (preventive service only) is associated with an individual’s chronic condition/risk status.

#### Oral health and chronic disease risk(s)

Three risk factors (available from the patient intake form completed in triage) of oral health disease and chronic disease risk(s) were utilized: 1) high blood pressure readings (pre-hypertensive, stage 1 hypertension, stage 2 hypertension, or isolated hypertension), 2) tobacco use, and 3) diabetes. Specifically, participants were asked 1) if they had ever been diagnosed by a physician with diabetes and 2) if they use tobacco. Tobacco use was defined as current use when a participant responded in the affirmative to the question. Additionally, blood pressure measures were collected from medical pre-screening.

The oral health and chronic disease risk(s) variable was categorized into three exclusive groups: “none” if the participant had no oral health and chronic disease risk(s), “one risk only” if the participant had only one health risk, and “two risks” if the participant had two health risks. One initial high blood pressure reading does not imply high blood pressure diagnosis or hypertension; yet, having hypertension may predispose individuals to certain chronic conditions [19]. Therefore, we included this measure in our analysis of the MOM participant profile. Some participants did report a high blood pressure diagnosis in their health history record, but the total “n” was not sufficient to include in our analysis plan.

### Statistical analysis

Descriptive statistics were calculated using SPSS (version 23) to assess frequencies and percentages of participant sociodemographics, oral health and chronic disease risk(s), and dental services. We used chi-square tests to determine the associations between 1) oral health and chronic disease risk(s) and dental services; and 2) oral health and chronic disease risk(s) and participant characteristics. A multivariate regression model was conducted to determine if participants with chronic conditions/risk(s) were more likely to receive certain dental services.

## Results

### Participant characteristics

Approximately 49% of the participants self-identified as black (*n* = 494), followed by Hispanic (*n* = 231, 22.9%), and white (*n* = 139, 13.8%). The majority of the participants were women (*n* = 623, 62.0%), and 38.0% (*n* = 377) were 18–34 years of age (Table 1). Slightly over 7 % of participants were 65 years or older. Approximately two thirds of the sample reported having one or more chronic conditions/risk(s) – diabetes, high blood pressure reading, and tobacco use (one chronic condition/risk only, *n* = 463, 46%; two chronic conditions/risks, *n* = 203, 20.2%). Approximately 10.9% (*n* = 110) reported diabetes diagnosis, 16.4% (*n* = 165) reported high blood pressure and 16.3% (*n* = 164) were tobacco users.

**Table 1:** Sample characteristics and summary of dental services: preventive care, and treatments at 2014 Mid-MD MOM (*N* = 1007)

Characteristics	% (N)
*Race & Ethnicity*	
White	13.8% (139)
Black	49.1% (494)
Hispanic	22.9% (231)
Other	5.8% (58)
Unreported	8.4% (85)
*Gender*	
Male	38.0% (382)
Female	62.0% (623)
*Age*	
18–34	38.0% (377)
35–49	30.2% (300)
50–64	24.5% (243)
65+	7.3% (72)
Chronic Condition(s) & Risk(s)	
*Diabetes*	
Yes	10.9% (110)
No	89.1% (897)
*High Blood Pressure Diagnosis*	
Yes	16.4% (165)
No	83.6% (843)
*Tobacco Use*	
Yes	16.3% (164)
No	83.7% (843)
*High Blood Pressure Reading*	
Normal	19.2% (194)
Pre-hypertensive	13.7% (138)
Hypertension (Stage 1, Stage 2, Isolated hypertension)	47.0% (474)
*Chronic Condition & Risks* ^*a*^	
None	33.9% (341)
Only 1chronic condition/risk	46.0% (463)
2 chronic conditions/risks	20.2% (203)
Preventive Services	
*Oral Hygiene Instruction*	
Yes	34.2% (344)
No	65.8% (663)
*Fluoride Varnish*	
Yes	7.1% (71)
No	92.9% (936)
*Adult Prophylaxis*	
Yes	26.7% (269)
No	73.3% (738)
Treatment Services	
*Full Mouth Debridement*	
Yes	16.7% (168)
No	83.3% (839)
*Restorative*	
Yes	35.9% (362)
No	64.1% (645)
*Endodontics*	
Yes	5.2% (52)
No	94.8% (955)
*Oral Surgery*	
Yes	13.5% (136)
No	86.5% (871)
No. of Combined Preventive and Treatment Services Delivered	
*No services*	33.4% (336)
*Preventive only*	10.3% (104)
Preventive & treatment	13.3% (134)
1 or more treatment only	43.0% (433)

^a^Chronic Condition & Risk is defined as including tobacco use, high blood pressure reading (Pre-hypertensive, Stage 1, Stage 2, & Isolated hypertension), and/or diabetes

Being Hispanic was associated with higher likelihood of having only one chronic condition/risk, compared with other racial and ethnic groups (Table 2). As noted, Hispanic participants (*p* = .004, *n* = 113, 48.9%) had the highest percentage of having only one chronic condition/risk. Whereas, white participants (*p* = .004, *n* = 42, 30.2%) had the highest percentage for having two chronic conditions/risks compared to other racial and ethnic groups. Men were more likely than women to have only one chronic condition/risk (*p* = .001, *n* = 189, 49.5%) and two chronic conditions/risks (*p* = .001, *n* = 90, 23.6%). Conversely, women were more likely than men to have no chronic conditions/risks (*p* = .001, *n* = 236, 37.9%).

**Table 2:** Associations of 2014 Mid-MD MOM patients’ characteristics by chronic condition(s) and risk(s)

		Chronic Condition(s) & Risk(s)	
	None	Only 1 Chronic Condition/Risk	2 Chronic Conditions/ Risks
Characteristics			
*Race & Ethnicity*	p = .004**		
White	26.6% (37)	43.2% (60)	30.2% (42)
Black	32.6% (161)	45.7% (226)	21.7% (107)
Hispanic	39.8% (92)	48.9% (113)	11.3% (26)
Other	36.2% (21)	46.6% (27)	17.2% (10)
Unreported	35.3% (30)	43.5% (37)	21.2% (18)
*Gender*	p = .001***		
Male	27.0% (103)	49.5% (189)	23.6% (90)
Female	37.9% (236)	44.0% (274)	18.1% (113)
*Age*	p < .001***		
18–34	41.6% (157)	44.0% (166)	14.3% (54)
35–49	35.0% (105)	47.0% (141)	18.0% (54)
50–64	22.6% (55)	49.8% (121)	27.6% (67)
65+	20.8% (15)	44.4% (32)	34.7% (25)

**p < 0.01; ***p < 0.001

Participants 18–34 years of age (*p* < .001, *n* = 157, 41.6%) had the highest percentage of having no chronic conditions/risks while those who were 65 years of age and older (*p* = .001, *n* = 25, 34.7%) had the highest percentage for two chronic conditions/risks.

### Dental services delivered

Among the preventive dental services delivered, oral hygiene instruction was the most common (*n* = 344, 34.2%) (Table 1). The most common dental treatment service was restorative (*n* = 362, 35.9%). Over a third (*n* = 433, 43.0%) of the participants received one or more treatments, but did not receive any preventive services, whereas 10% (*n* = 104, 10.3%) of the sample only received preventive services.

While 1007 individuals registered, after a participant was assessed in medical triage, the participant may not have received any service because he/she was deemed medically ineligible as a result of their current medical condition, required pre-medication, or there was not enough time to provide the necessary dental service (33.4%, *n* = 336).

### Associations of chronic condition(s) and risk(s) with dental services

Bivariate associations (Table 3) revealed having one or more chronic conditions/risks was significantly associated with the likelihood of receiving restorative and oral surgery services (specifically, tooth extraction). Participants with only one chronic condition/risk were significantly more likely to receive a restoration (*p* < .001, 44.1%) compared to participants with none or two chronic conditions/risks. Participants had higher probabilities of an extraction when they had more chronic conditions/risks. These chronic conditions/risks were not significantly associated with the likelihood of receiving a preventive service or endodontics.

**Table 3:** Associations between 2014 Mid-MD MOM patients’ chronic condition(s) and risk(s) with dental services

	Dental Services				
	Preventive Only	Full Mouth Debridement	Restorative	Endodontics	Oral Surgery
Chronic	p = .02*	p < .001***	p < .001***	*p* = .83	p < .001***
Condition(s) & Risk(s)					
None	18.5% (63)	9.7% (33)	28.7% (98)	4.7% (16)	7.3% (25)
Only 1 factor	27.0% (125)	20.7% (96)	44.1% (204)	5.6% (26)	15.8% (73)
2 factors	24.6% (50)	19.2% (39)	29.6% (60)	4.9% (10)	18.7% (38)

*p < 0.05; ***p < 0.001

Chronic condition(s) and risk(s) is defined as including tobacco use, high blood pressure readings (Pre-hypertensive, Stage 1, Stage 2, & Isolated hypertension), and/or diabetes

We used a multivariate logistic regression model to determine if participants with one or more chronic conditions/risks were more likely to receive certain dental services, specifically more invasive services (Table 4). After controlling for participant characteristics, those who had only one chronic condition/risk were more likely to receive a full mouth debridement (only one condition/risk: OR = 2.33, 95%, CI = 1.51–3.58, *p* < .001; two conditions/risks: OR = 2.08, 95%, CI = 1.23–3.50, *p* = .001) and an extraction (only one condition/risk: OR = 2.40, 95%, CI = 1.48–3.90, *p* < .001; two conditions/risks: OR = 3.12, 95%, CI = 1.78–5.46, *p* < .001). Respondents with one condition/risk were also significantly more likely to receive a restorative procedure compared to respondents with no risk factor (OR = 2.05, 95% CI = 1.51–2.80). Likelihood of receiving any restorative procedures were similar among respondents with two or more conditions/risks and those with no condition/risk.

**Table 4:** Multivariate logistic regression of 2014 Mid-MD MOM patients’ chronic condition(s) and risk(s) by dental services controlling for patient characteristics

	Dental Treatment				
	Preventive Only	Full Mouth Debridement	Endodontic	Restorative	Oral Surgery
	OR, 95% CI	OR, 95% CI	OR, 95% CI	OR, 95% CI	OR, 95% CI
Chronic Condition(s) & Risk(s) (Ref = None)					
Only 1 chronic condition/risk	1.63 (1.15–2.33)*	2.33 (1.51–3.58)***	1.32 (.69–2.54)	2.05 (1.51–2.80)***	2.40 (1.48–3.90)***
2 chronic conditions/risks	1.46 (.93–2.29)	2.08 (1.23–3.50)**	1.23 (.53–2.85)	1.13 (.76–1.69)	3.12 (1.78–5.46)***

* *p* < 0.05; **p < 0.01; ***p < 0.001

## Discussion

Our study reports findings of the relationship between having multiple chronic conditions/risks and receiving invasive (oral surgery-tooth extractions) and other dental services of participants from the 2014 Mid-Maryland Mission of Mercy (MOM) and Health Equity Festival (HEF). While participants primarily attended the event to receive dental care; nonetheless, our analyses revealed that a third of them had one or more chronic conditions and lifestyle risk behaviors and that these conditions were more prevalent in older than younger participants. In addition, those with two or more chronic conditions and risks were more likely to have oral surgery than those with one condition/risk.

By incorporating a HEF, based on the three key elements necessary when implementing a “community-driven” initiative aimed at achieving health equity [14], into the traditional MOM format, we helped participants identify other health concerns and treatment options – namely, emergency care, coordinated care, oral healthcare services, and primary care follow-up. Given the relationship between chronic risks like diabetes, high blood pressure, and oral health diseases, these conditions warrant the need for behavioral changes such as a healthy diet/nutrition, smoking cessation, practicing oral hygiene, and using fluoride [20, 21].

The dental service decisions were often based on the “most” urgent need identified by the participant, as well as availability of appropriate dental care providers at the MOM event. Consequently, we recognize that all of the services delivered may not have been the result of participants’ oral health and/or general health behavior but were due to limited time and resources. Due to varying Medicaid coverage for adult dental care [22], we recognize that certain oral diseases are often untreated, leading to infections, pain, and the inability to eat, which are all associated with more expensive and invasive, yet preventable dental services. Thus, we explored how an individual’s general health status impacts receipt of an invasive or non-invasive dental health service. Such findings provide evidence regarding the need for comprehensive, combined (general + oral) health services.

Our analysis of the data revealed additional links between chronic conditions/risks and oral healthcare; specifically as it relates to dental services. Prior research examined oral health behaviors such as hygiene practices [23, 24] and disease [25]. Our study examined associations with dental services delivered in a MOM community dental setting. An individual having one or more chronic conditions or oral health risk increased their likelihood of receiving three of the four dental services, preventive, full mouth debridement, restorative, and extractions. Still, an individual with more than one chronic condition and/or risk had a higher chance of an extraction than full mouth debridement.

In 2015, the American Dental Association conducted an assessment of self-reported oral health status, attitudes, and dental care utilization among Maryland adults, titled *Oral Health and Well-Being in Maryland* [26]. According to the report, 25% of low income adults had difficulty accessing a dentist, 31% of middle income adults and 41% of high income adults were fearful of visiting the dentist [26]. Our findings reveal that many MOM participants are in need of coordinated care for their primary and dental care needs. Although some of these services were based on urgent need, our findings demonstrate that certain services rendered [e.g., restorative or oral surgery services (specifically, tooth extraction) due to infections or deterioration] were preventable if the participant had access to comprehensive dental care.

It is also important to note that although the majority of the MOM participants were Hispanic and African American, white participants were more likely to have two chronic conditions/risks compared to all other racial and ethnic groups. This may be attributed to our sample not being representative of the general U.S. population. In addition, other factors such as income, education level, health insurance and level of health literacy may contribute to these findings.

We have considered several alternatives for inclusion in future MOM and HEF events: 1) reposition access to the HEF and integrate it into the flow of participant registration and medical triage; 2) allow some participants to enter the HEF prior to receiving their dental service, while others access it after their care; allowing for efficient data collection that neither overburdens the participant nor creates a barrier for the healthcare providers delivering services; 3) include self-rated health status, healthcare coverage, education status, physical activity frequency, and body mass index within the dental record; and 4) create data platforms to merge participant profiles with health and social service referral systems, which could be linked to appropriate HEF vendors who provide effective follow-up for oral health and primary healthcare to MOM participants, such as federally qualified health centers. These alternatives could ensure that MOM attendees are linked to ongoing primary care and dental services located in close proximity to their neighborhood.

## Conclusion

Our findings reveal that attendees at the 2014 Mid-Maryland Mission of Mercy and Health Equity Festival were living with multiple risk factors or chronic conditions and in dire need of both combined (general + oral) health services. While such events will not solve the dental and general health needs of all Maryland residents, it is important to understand the role that an initiative such as MOM can play in addressing the overlapping chronic conditions such as diabetes with oral diseases. This assessment challenges us to consistently examine how we develop and implement these public health events and how we design their affiliated services. This will allow for streamlining future operations, and tailoring this type of forum with its related health equity activities for more effective follow-up and on-going care after a MOM event has ended. In the absence of a comprehensive policy solution to the oral health crisis, we have a moral obligation to alleviate human suffering with temporary solutions like MOM events. Given the frequency of Mission of Mercy dental clinics and the continued demand for the charitable services they provide, we must design them in a manner with a shared vision to eliminate oral health disparities and achieve health equity.

## Abbreviations

HEF: Health Equity Festival
IRB: Institutional Review Board
M-CHE: Maryland Center for Health Equity
MOM: Mission of Mercy
